# Editorial: The Epidemiology, Diagnosis and Prevention of Infectious Diseases in Livestock

**DOI:** 10.3389/fvets.2021.840635

**Published:** 2022-01-24

**Authors:** Satoshi Sekiguchi, Anuwat Wiratsudakul, Van Giap Nguyen

**Affiliations:** ^1^Department of Veterinary Science, Faculty of Agriculture, University of Miyazaki, Miyazaki, Japan; ^2^Faculty of Veterinary Science, Mahidol University, Nakhon Pathom, Thailand; ^3^Faculty of Veterinary Medicine, Vietnam National University of Agriculture, Hanoi, Vietnam

**Keywords:** epidemiology, diagnosis, infectious diseases, prevention, livestock

The infectious disease in livestock plays a vital role in the economy and food security of many developing countries. An approach in veterinary epidemiology to animal infectious diseases focuses on controlling and managing disease in the livestock population. Additionally, the risk of transmission and spread of emerging diseases is increasing because of intensive human traffic. In such circumstance, those outbreaks cause severe economic losses when the livestock industry become affected. Therefore, a major component of animal disease policies and disease management strategies are a viable prevention measure. However, the same prevention doesn't work everywhere due to strategies has changed over time. An emergency preparedness needs to do before outbreaks were reported.

This Research Topic yielded 45 articles of which 10 were brief research report, 30 original research articles, one clinical trial, one opinion, two systematic reviews, and one review, involving 321 authors from 17 countries. The two areas of research cover by this Research Topic are included: (i) infectious disease and control or eradication in livestock; (ii) development of diagnosis, monitoring, and surveillance program.

A first line of research includes the infectious disease and control or eradication in livestock. Intervention can vary depending on clinical fields and local circumstances. In this part, their works presents the appropriate framework needed for strengthening a surveillance program in each region. Serology has been performed for many years and the present of antibody refer as a marker to identify reservoir that may boost the outbreaks. In their work, Sun et al. report of *Chlamydia* seroprevalence in domestic, black-boned sheep and goats in southwest China. Involving same methods, Ullah et al. also reported anti-*Brucella* antibodies in sheep and goats at these livestock farms in Punjab, Pakistan. Furthermore, serological cross-reactivity was detected between alphaherpesvirus-2 and alphaherpesvirus−1 in a calf located in Central Italy by Petrini et al. However, this method is only required for early diagnosis and infection occurring in previously vaccinated animals may not be detectible. To overcome the limitation mentioned above, the molecular epidemiology of infectious diseases is also discussed in this Research Topic. For examples, isolation and characterization of a porcine transmissible gastroenteritis coronavirus in Northeast China by Yuan et al.; epidemiology of bovine tuberculosis in Ethiopia by Tulu et al. Similarly, an interesting work led by Hamada et al. described the new investigation on molecular prevalence of bovine leukemia virus in Egyptian cattle. In an article by Shi et al., they revealed bovine pestivirus isolates from cattle have extensive genetic variations in Central China.

There were still many studies highlighted the evaluation of the prevention of infectious disease in livestock. As the case in Vietnam, Mai et al. find out the possible risk factors for transmission pattern of PEDV in an endemic region. They indicated that there have three of these 29 variables such as farrow-to-wean production type, distance from the farm to the slaughterhouse, and the presence of chickens on site. Recently, Makita et al. also examined the decision-making process for farm biosecurity among livestock farmers through elevated attitudes and self-efficacy. An integrated community-based intervention is also applied for control and prevention of epizootic lymphangitis in mules, Bahir Dar, Ethiopia, which was reported by Duguma et al.

This Research Topic is further investigating the role of wildlife in the epidemiology of infectious diseases. In addition, the identification of microorganisms by vectors has been useful for identifying reservoirs and verifying modes of transmission. An outcome of the papers by Chang et al. indicated pigeons play an important role in transmission pattern of Pigeon paramyxovirus type I. Works reporting the evidence of wild animals, as the case in Korea, wild leopard cat and Asian badger may spread feline parvovirus and its related viruses, which was reported by Kim et al. or in Germany, Q fever is presented by Winter et al. in ruminant. In particular, the work led by Shimizu et al. shown that infected wild boars are a major source of infection for the current classical swine fever occurred in Japan. A retrospective survey by Zhou et al. indicated that the blue fox's outbreak of abortions was derived from brucellosis which caused by *B. melitensis* strain. Moreover, a survey by Kivali et al. identified trypanosome species in cattle and their spatial distribution in western Kenya.

It was well-known that those novel pathogens are also responsible for emerging infectious diseases. A work led by Yu et al. shown that, as in goat, the impact of endogenous and exogenous factors affecting susceptibility to orf virus will likely reflect the host's specific in term of a particular strain such as NZ7-like orf viruses. An additional paper by Shimizu et al., presented the report of genetic variability of orf viruses in Japan, which can be spread to sheep or farmers and can be transmit to Japanese serows. Along this line, Su et al. showed that a coinfection porcine astrovirus with PEV and GARV in diarrheic piglets in China. Furthermore, Getah virus was reported by Ren et al. in southern China.

A second related aspects of research that drew the development of diagnosis, monitoring, and surveillance program. The application of new innovative diagnostic technologies for rapidly detect of pathogens is required for limit the economic impact of emerging infectious animal diseases. The desirable characteristics are fast, simple, cost-effective, highly sensitive, and specific. In these lines, a rapid detection of PCV2 and direct identification of PCV2 genotypes from clinical samples was developed by Wang, Song, Shin, Kim et al., examined the performance of two newly available methods, multiplex real-time PCR assay and PCR-reverse blot hybridization assay. These techniques can also yield results in 2–3 h for differentiate between the PCV2 genotypes and detect PCV2 from clinical specimens. Continuing with the search of novel diagnostic, Wang, Song, Shin, Choi et al. also highlighted that using the new clinical molecular assay for diagnosis in porcine cytomegalovirus (PCMV) can be used to screen with dramatic reduction in false positives and negatives. Besides, Arrieta-Villegas et al. examined the applicability of the P22 antigen complex as a complementary tool for TB diagnostics in goats with a cost-effective alternative. The review by Rodríguez-Hernández et al. also highlight application of Volatilome analysis to the diagnosis of mycobacteria infection in livestock, which was fulfills part of the mycobacterial diagnosis requirements.

The remaining works further highlighted the novel methods such as one-tube nested RT-PCR which required ~1.5 h for completion for the characterizing pathogen responsible for porcine cytomegalovirus infection (Wang, Song, Shin, Choi et al.); or multiplex PCR is design to provide a rapid, specific and sensitive detection method for the identification of four pathogenic bacteria in minks (Li et al.) and thermal image scanning for the early detection of fever induced by highly pathogenic avian influenza virus infection in chickens and ducks (Noh et al.). Similarly, a procedure for partial or full genome sequencing of peste-des-petits-ruminants virus is described by Torsson et al. The use of a portable laboratory such as miniPCR and MinION to the field and to the production of a full genome with the results just 24 h of collection. On the other hand, Ruggeri et al. indicated that the newly scoring system could be used to exam pathological lesions in the respiratory tracts in porcine respiratory disease complex.

Taken together, all the published papers in this Research Topic reflects that a combined effort across borders is needed to control infectious diseases in livestock on such epidemiology, diagnosis, and prevention and coordinated action around the globe required to fill gaps in the literature regarding control and eradication programs for infectious diseases.

## Author Contributions

VN and SS wrote the first part of the infectious disease and control or eradication in livestock. AW wrote the second part of the development of the diagnosis, monitoring, and surveillance program. All authors contributed to conception, design of the project, manuscript revision, read, edited, added sections, provided critical comments of the manuscript, and agreed on the submitted version.

## Conflict of Interest

The authors declare that the research was conducted in the absence of any commercial or financial relationships that could be construed as a potential conflict of interest.

## Publisher's Note

All claims expressed in this article are solely those of the authors and do not necessarily represent those of their affiliated organizations, or those of the publisher, the editors and the reviewers. Any product that may be evaluated in this article, or claim that may be made by its manufacturer, is not guaranteed or endorsed by the publisher.

